# Monoparametric high-resolution diffusion weighted MRI as a possible first step in an MRI-directed diagnostic pathway for men with suspicion of prostate cancer

**DOI:** 10.3389/fonc.2023.1102860

**Published:** 2023-01-31

**Authors:** Jeroen Sebastiaan Reijnen, Una Ryg, Jon B. Marthinsen, Irina Schönhardt, Therese Seierstad, Knut H. Hole

**Affiliations:** ^1^ Department of Radiology, Sørlandet Hospital Trust, Kristiansand, Norway; ^2^ Institute of Clinical Medicine, University of Oslo, Oslo, Norway; ^3^ Division of Radiology and Nuclear Medicine, Radiumhospitalet, Oslo University Hospital, Oslo, Norway; ^4^ Department of Pathology, Sørlandet Hospital Trust, Kristiansand, Norway

**Keywords:** prostatic neoplasms, magnetic resonance imaging, follow-up studies, prospective studies, algorithms (MeSH term)

## Abstract

**Purpose:**

To explore if a high-resolution diffusion weighted MRI sequence (DWI-only) could be used as a first step in an MRI-directed diagnostic pathway.

**Methods:**

Prospective single center study that between December 2017 and August 2018 included 129 consecutive patients with suspicion of prostate cancer into a PI-RADS-based MRI-directed diagnostic pathway. All patients had multiparametric MRI (mpMRI). Based on only the transversal high-resolution DWI images two consultant radiologists prospectively categorized the findings as positive, equivocal, or negative for clinically significant cancer. The radiologists then interpreted the mpMRI and assigned a PI-RADS score. A third independent reader retrospectively categorized the DWI-only exams without access to the mpMRI. The interpretations of DWI-only were compared to the PI-RADS classification from mpMRI and the histopathology from the biopsies. Non-biopsied patients were followed in a safety net monitoring for 56 months.

**Results:**

Based on DWI-only, 29 (22.5%) of the exams were categorized as negative, 38 (29.5%) as equivocal and 62 (48.1%) as positive. Of the 56 patients with PI-RADS 4-5 at mpMRI, 55 were also categorized as positive at DWI-only. All patients diagnosed with clinically significant cancer were identified using DWI-only. 56 months of safety net monitoring did not reveal any clinically significant cancers among patients with exams categorized as negative or equivocal. There was high inter-reader agreement on positive findings, but less agreement on negative and equivocal findings.

**Conclusions:**

In this concept study, the monoparametric DWI-only identified all patients with clinically significant cancer in a mpMRI-directed diagnostic pathway.

## 1 Introduction

A PI-RADS based MRI-directed pathway is recommended for the assessment of men with first time suspicion of prostate cancer (1, 2), but there are several obstacles that limit its incorporation into clinical routine. PI-RADS requires multiparametric MRI (mpMRI) with the use of contrast medium, which increases costs and may not offer additional benefit for the detection of clinically significant cancer (3–6). In addition, there is a shortage of experienced radiologists and limited access to MRI scanners. The PI-RADS steering committee states that widespread implementation of mpMRI is therefore at risk (6).

In addition to its role in the assessment of suspected cancers, prostate MRI is advocated as a monitoring tool in active surveillance of cancers for which treatment is deferred (7, 8). Moreover, there is a growing interest in prostate cancer screening with MRI. These developments place increasing strain on MRI accessibility and costs and generate a need for simplifying and shortening the examination (9).

The PI-RADS committee has recently published a narrative review discussing biparametric MRI (bpMRI) without contrast medium (6). They suggested that possible approaches could be to implement bpMRI given certain prerequisites, or to select men to MRI with contrast medium based on clinical risk parameters. However, they concluded that higher-quality data are needed before the committee can make evidence-based recommendations (6). Parallel to the study of van der Leest et al. (5) we set out to explore the performance of a fast MRI protocol. Not with the intention to replace mpMRI, but as a possible first step to select men that could benefit of proceeding to mpMRI. Small field of view (zoomed) echo planar imaging (EPI) has the potential to generate DWI images with high tumor to background contrast and high spatial resolution (10).

The aim of this concept study was to explore the idea of using a fast MR examination as a first step to triage patient to either monitoring or full mpMRI. We compared the prospective interpretation of a high-resolution DWI-only to the subsequent interpretation of the full mpMRI and to the outcome of a PI-RADS based MRI-directed diagnostic pathway with a median follow-up of 56 months.

## 2 Materials and methods

### 2.1 Study cohort

In Norway, each county hospital serves a geographically confined population. For our hospital, this population consists of 187 000 people. In order to be examined or treated in the specialized health care, men with suspected prostate cancer within this population are referred by their GP to the urology department at our hospital. Criteria for the GP for such referral are defined in the standardized cancer patient pathway for prostate cancer in Norway, and include a general clinical examination, family history, digital rectal examination (DRE) and PSA measurements. Elevated PSA levels are defined as >2.5 ng/ml for men below 50 years, >3.5 ng/ml for men between 50-59 years and >4.0 ng/ml for men of 60 and older. Pathologic DRE is referred independent of PSA value. When DRE is normal, PSA is repeated to rule out infection (measured twice with an interval of 3 weeks). The urologist initiates the diagnostic pathway when there is reasonable suspicion of prostate cancer based on clinical information from the GP. Between December 2017 and August 2018, 129 consecutive biopsy-naïve men were enrolled in our institutional PI-RADS-based MRI-directed diagnostic pathway (11). The median age was 64.2 years and the median prostate specific antigen (PSA) was 6.7 ng/ml (**Table 1**). The institutional review board approved the study and waived the need for informed consent.

**Table 1:** Patient demographic and clinical data. Data are given as median and range.

	All	DWI-only negative	DWI-only equivocal	DWI-only positive
Age (years)	64 (47-76)	64 (47-75)	64 (53-74)	66 (47-76)
PSA (ng/ml)	6.7 (0.7-40)	5.1 (0.7-18)^*#^	6.9 (1.8-22)^*^	7.9 (1.6-40)^#^
Prostate volume	46 (16-150)	61 (20-150)^$^	58 (21-145)^&^	36 (16-105)^$&^
PSA density	0.15 (0.02-1.95)	0.08 (0.02-0.23)^@#^	0.14 (0.05-0.45) ^@%^	0.21 (0.05-1.95)^#%^

The p-values for the comparison between two different groups are shown with different symbols: ^*^p = 0.03, ^#^p < 0.001, ^$^p = 0.002, ^&^p = 0.002, ^%^p < 0.001, ^@^p < 0.003.

### 2.2 MRI acquisition

All MRI examinations were performed using a 3T Siemens MAGNETOM Skyra MRI scanner and phased-array coil. The patients voided the rectum with a cleansing enema (toilax 10 mg/5 ml bisakodyl, Orion Corporation) and were given 2 mg butylskopolamin (Boehringer Ingelheim) intravenously and 1 mg glucagon intramuscularly to reduce peristalsis according to the institutional protocol.

All patients had mpMRI consisting of morphological T1- and T2-weighted images, DWI, and DCE images. The MRI sequences and acquisition parameters (**Table 2**) comply with the current technical requirements from PI-RADS (12, **4**, 13). DCE imaging was performed after i.v. injection of gadoterate meglumine (Dotarem, Guerbet LLC) at a dose of 0.1 mmol/kg body weight at a rate of 2 ml/s followed by a 20 ml saline flush. High-resolution DWI was acquired with the zoomed EPI-based sequence from Siemens (ZOOMit) with b values of 0 and 800 mm2/s and calculated b1400. The ADC maps were calculated from b0 and b800.

**Table 2:** The MRI scan protocol.

Acquisition parameters	T2W TSE	DWI ZOOMit	T2W SPACE	DCE	T1W post Gd
MRI sequence	2D SE	SE-EPI zoomed	3D SE	3D Spoiled GE-Dixon	3D Spoiled GE-Dixon
Acquisition plane	sag/tra/cor	tra	tra	tra	cor
Echo time (ms)	99	69	103	1.28 and 2.51	2.46 and 3.69
Repetition time (ms)	2000 (DE)	3800	1240	4.27	5.45
Flip angle	150		125	10	10
Slice thickness (mm) Acquired Interpolated	3	3	1.42 1.0	3.30 3.00	2.36 1.50
Slice gap (mm)	0	0.8	0	0	0
Number of excitations	1/3/1	16	1.4	1	1
In-plane resolution (mm x mm) Acquired Interpolated	0.69x0.69/0.63x0.63/0.69x0.69 0.34x0.34/0.31x0.31/0.34x0.34	1.69x1.69 0.85x0.85	0.93x0.88 0.44x0.44	1.38x1.31 0.66x0.66	0.90x0.90 0.45x0.45
Echo train	16/14/16	44	100	NA	NA
Bandwidth (Hz/pixel)	295	1500	651	1080-890	870-810
FOV (mm x mm)	220x220/200x200/220x220	115 x 74	225x225	210x210	345x345
Matrix size (pixels x pixels) Acquired Interpolated	320 x 320 640 x 640	68x68 136x136	256x243 512x512	160x152 320x320	384x384 768x768
Parallell imaging acceleration factor^a^	1/2/1	1	3	2x2	2x2
Motion correction	no	no	no	yes	no
b-values (s/mm^2^)	NA	0-800; calculated b1400	NA	NA	NA
Time resolution (sec)	NA	NA	NA	10	NA
Acquisition time	2:46/4:42/2:46	5:17	5:19	4:34	1:28

T2W = T2 weighted MRI, TSE = turbo spin echo, DWI = Diffusion Weighted Imaging, DCE = Dynamic Contrast-Enhanced MRI, Gd = Gadolinium, SE = Spin Echo, EPI = Echo Planar Imaging, GE = gradient echo, DE = Driven Equilibrium, FOV = field of view, SPACE = 3D SE, DWI ZOOMit = zoomed EPI-based DWI. NA=Not Applicable

a Parallel imaging acceleration factor in more than one direction are given as (NxN).

### 2.3 MRI interpretation

Two consultant radiologists (J.S.R., J.B.M.) with 10 and 5 years of prostate MRI experience prospectively evaluated the MRI examinations, if in doubt in consensus.

**Figure 1:**
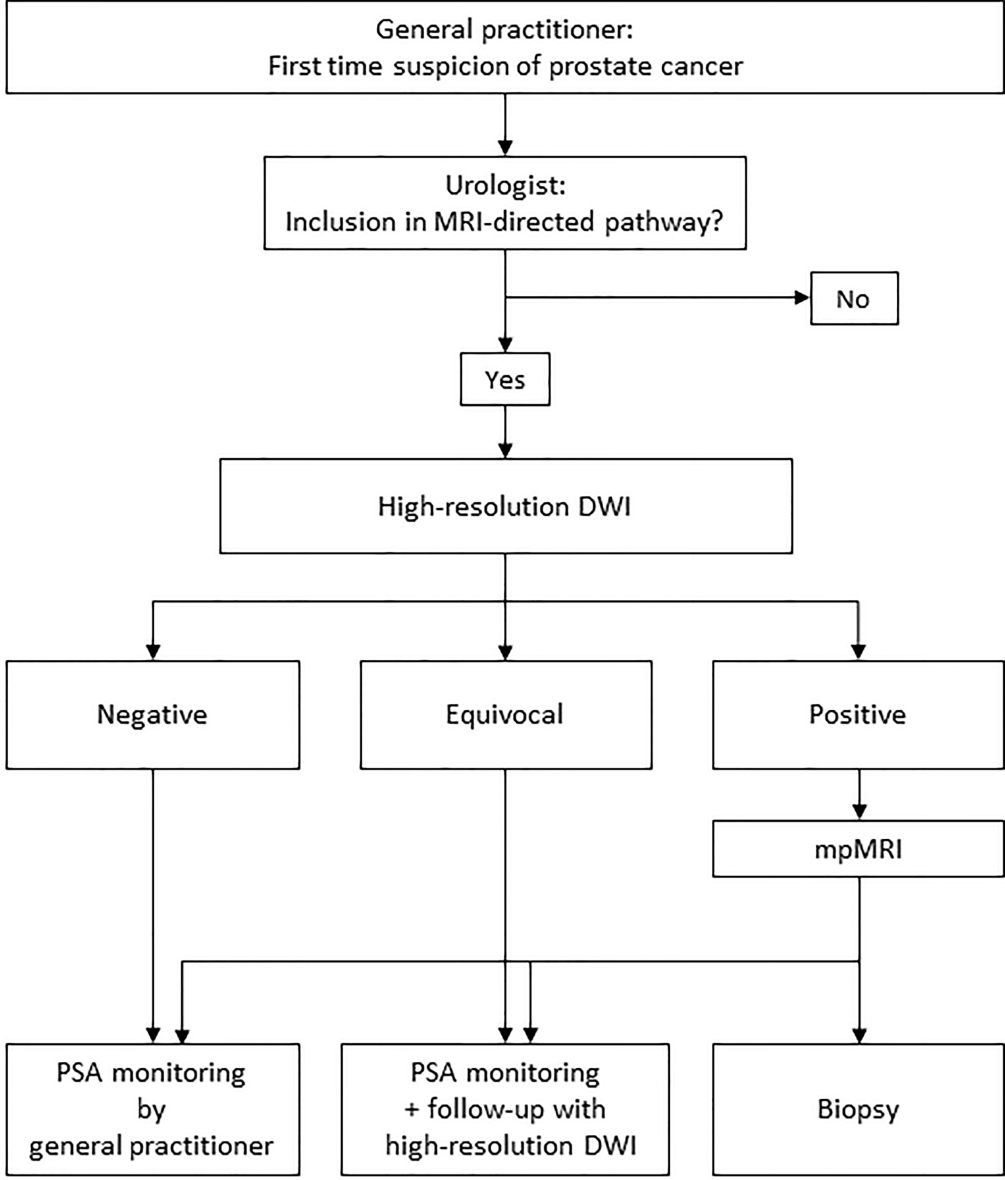
Illustration of the concept of an MRI-directed diagnostic pathway with high-resolution DWI-only as a first step. In a clinical setting, clinical parameters are also taken into consideration before ruling out biopsy in MRI negative cases.

Initially only the high-resolution DWI images were reviewed without access to any of the other mpMRI sequences, b0, b800, calculated b1400 and ADC were available. The interpretations were categorized into positive, equivocal, or negative for clinically significant cancer. The findings were interpreted with the assumption that positives would proceed to mpMRI, whereas equivocals and negatives would be referred to monitoring as illustrated in **Figure 1**.

**Figure 2:**
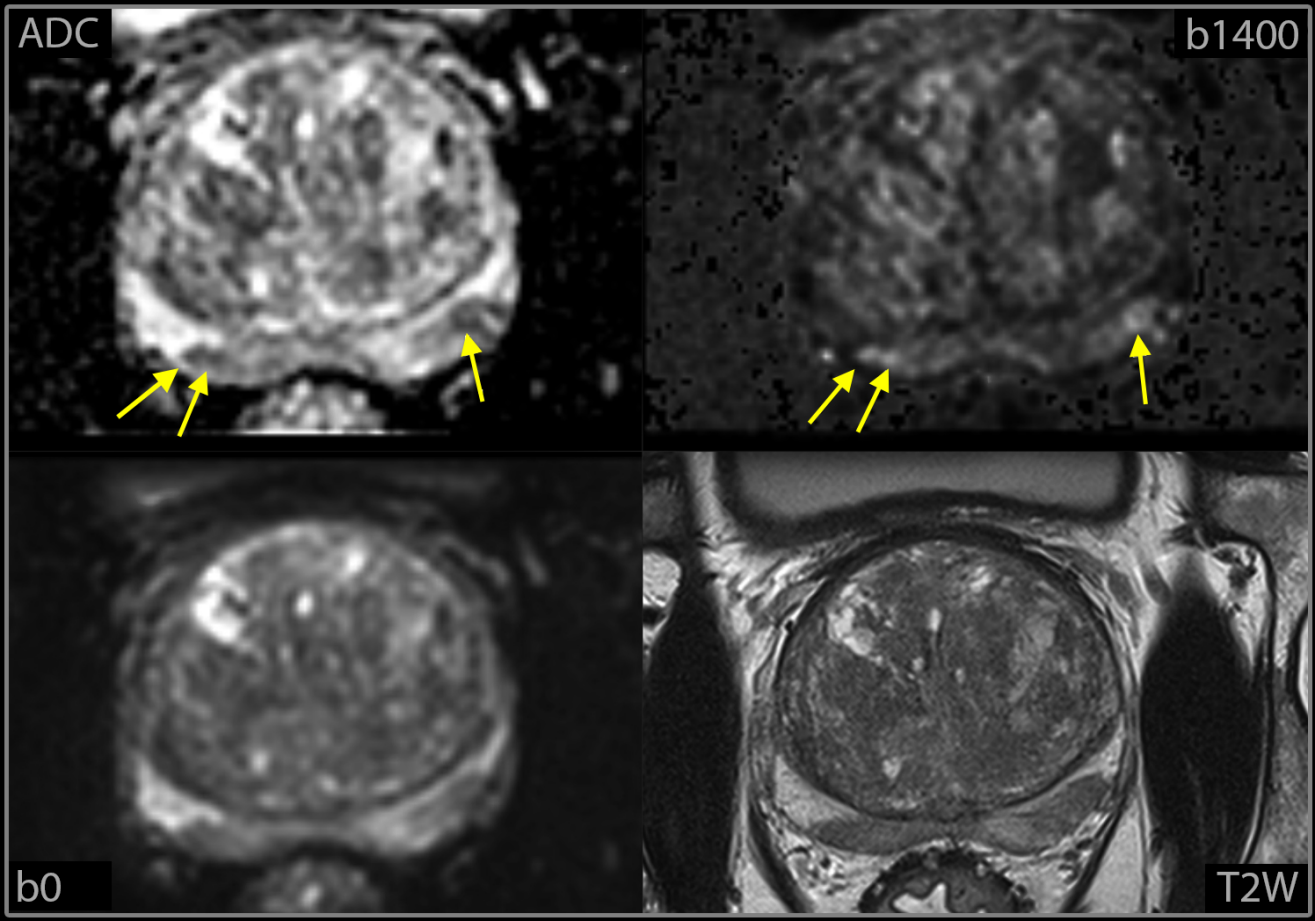
Example of small foci (yellow arrows) classified as equivocal at high-resolution DWI-only, with T2W sequence from mpMRI for comparison.

**Figure 3:**
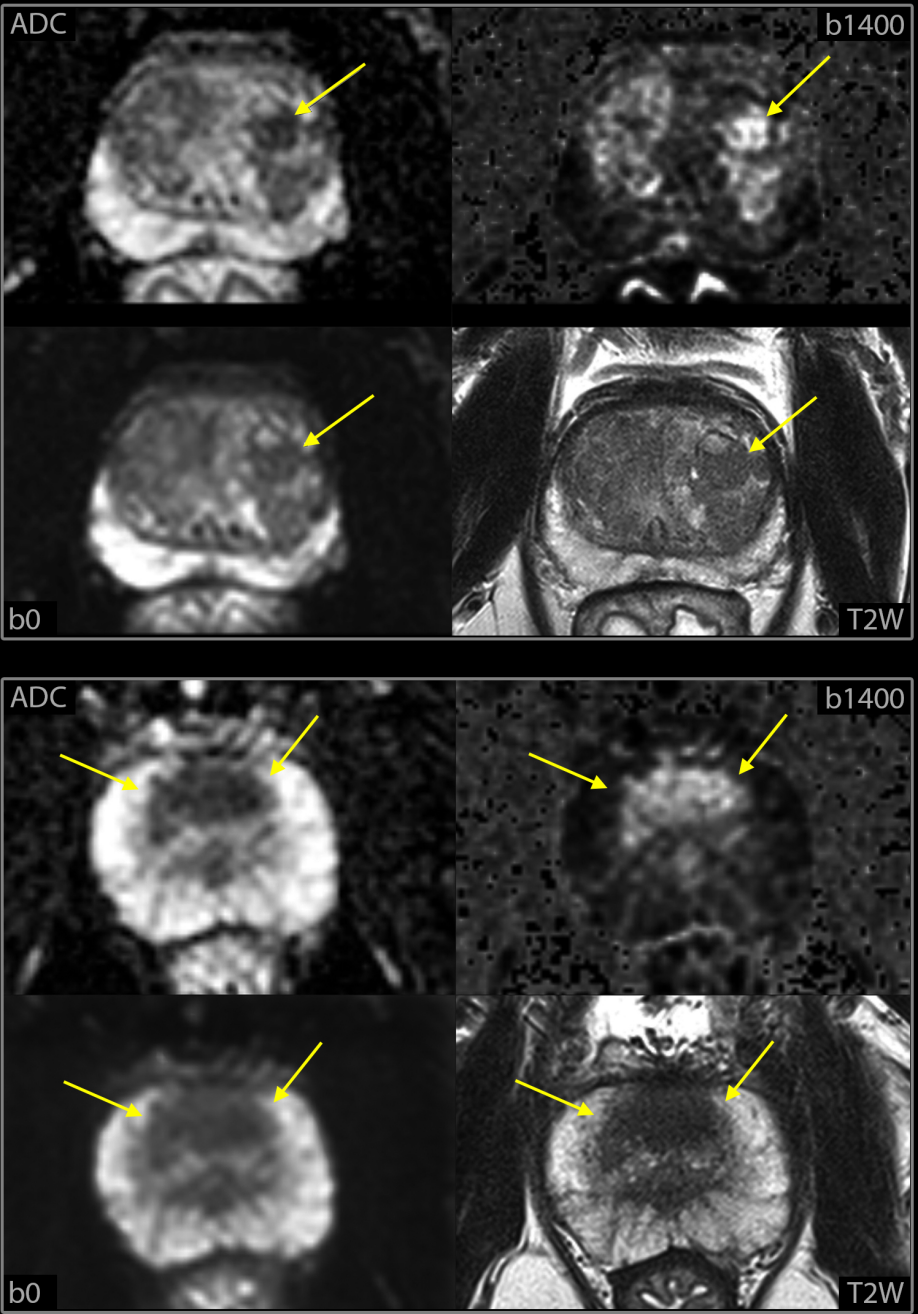
Example of findings in the transitional zone at high-resolution DWI-only, Highly cellular lesions (yellow arrows), at calculated b1400 images and ADC maps, were interpreted as hyperplastic nodules and categorized as negative if round and centrally located at b0 (upper panel) but categorized as positive if crescent shaped and anteriorly located (lower panel). T2W images from mpMRI are included to illustrate the similarity in the morphologic appearance with the b0 DWI images.

The radiologists recorded whether the findings were in the peripheral zone or in the transitional zone. In the peripheral zone they used the PI-RADS DWI criteria, but suspicious findings less than 3 mm in diameter were categorized as equivocal (**Figure 2**). In the transitional zone the T2W score is the dominant factor that determines the PI-RADS assessment, sometimes upgraded from 2 to 3 or from 3 to 4 based on a high DWI score (4). They primarily used the DWI score to evaluate findings in the transitional zone since spin-echo T2W was not available. Because DWI b0 is a T2W fat-saturated image, they also considered the morphological appearance on the DWI b0 images, before assigning a final score of positive, negative, or equivocal. **Figure 3** illustrates a case in which the morphological appearance at b0 downgrades the DWI score (upper panel), and a case in which the morphological appearance at b0 indicates a high T2W score (lower panel).

After categorizing the HR DWI only, the same two consultant radiologists (J.S.R., J.B.M.) interpreted the full mpMRI and assigned a PI-RADS score (11). The interpretation categories from DWI-only and the PI-RADS scores from mpMRI were prospectively entered into the institutional Prostate Cancer Quality Registry.

To assess whether another radiologist could achieve the same results, i.e. the generalizability of DWI-only, a third, independent reader (U.R.) with less experience (3.5 years) of prostate MRI retrospectively interpreted the high-resolution DWI without access to the other MRI sequences. This reader was instructed to categorize the findings according to the same criteria as the two other readers. Patient age and PSA value, but no other clinical information was available to the third reader.

### 2.4 Reference standards

We used two levels of reference standards, both the PI-RADS interpretation from mpMRI and the clinical outcome of our MRI-directed pathway (11). In this pathway, patients with PI-RADS 1-2 were not biopsied unless risk factors were present (PSA metrics, DRE findings, family history, comorbidity, and life expectancy). Systematic transrectal ultrasound (TRUS) biopsies were obtained for patients with risk factors. For patients with PI-RADS 3, the multidisciplinary team (MDT) decided whether to biopsy based on MRI and risk factors. Patients with PI-RADS 4-5 were as a rule referred to biopsy, either TRUS biopsies or MRI in-bore biopsies for small tumors and for sites difficult to access. All non-biopsied patients were followed for a median of 56 months (range 52-60) in a safety net monitoring regimen as described in our previous paper (11). In this regimen, non-biopsied patients with PI-RADS 1-2 were referred for follow-up by their GP, with instructions to contact the urologist at our institution if the PSA metrics increased above a threshold value. Non-biopsied patients with PI-RADS 3 were followed with PSA measurements, mainly at six-month intervals carried out by the hospital or the GP. Six of the non-biopsied patients later had repeat MRI(s). Two of these patients also had TRUS biopsies, one negative and one ISUP 1. It is important to point out that the public health care system in our region is organized so that all patients are referred back to our hospital exclusively. Clinically significant cancer was defined as Gleason score of at least 3 + 4 (ISUP grade group 2) (2, 14).

### 2.5 Data analyses

We used descriptive statistics to compare the results from DWI-only to PI-RADS from mpMRI and the clinical outcome of our MRI-directed diagnostic pathway. The five PI-RADS scores were simplified to PI-RADS 1-2, PI-RADS 3, and PI-RADS 4-5 for comparison with the three categories from high-resolution DWI: negative, equivocal, and positive. We performed a sub-analysis of the equivocal and positive DWI-only findings in the transitional zone because larger discrepancies with mpMRI could be anticipated due to the lack of the dominant T2W sequence.

The accuracy of DWI-only to detect clinically significant prostate cancer (ISUP ≥ 2) was calculated considering equivocal and negative cases as the same group, because in our concept both these groups would not proceed to biopsy but enter safety net monitoring. True positive cases are patients positive at DWI-only with ISUP ≥ 2 (biopsy). True negative cases are patients negative or equivocal at DWI-only and ISUP < 2 (biopsy) or not diagnosed with clinically significant cancer within the 56 months median follow-up.

Inter-reader agreement was assessed by calculating Cohen’s K.

Prostate volume from the DWI images was recorded and PSA density was calculated. The demographic and clinical data for the three DWI-only categories were compared using the Mann-Whitney U test.

## 3 Results

**Figure 4:**
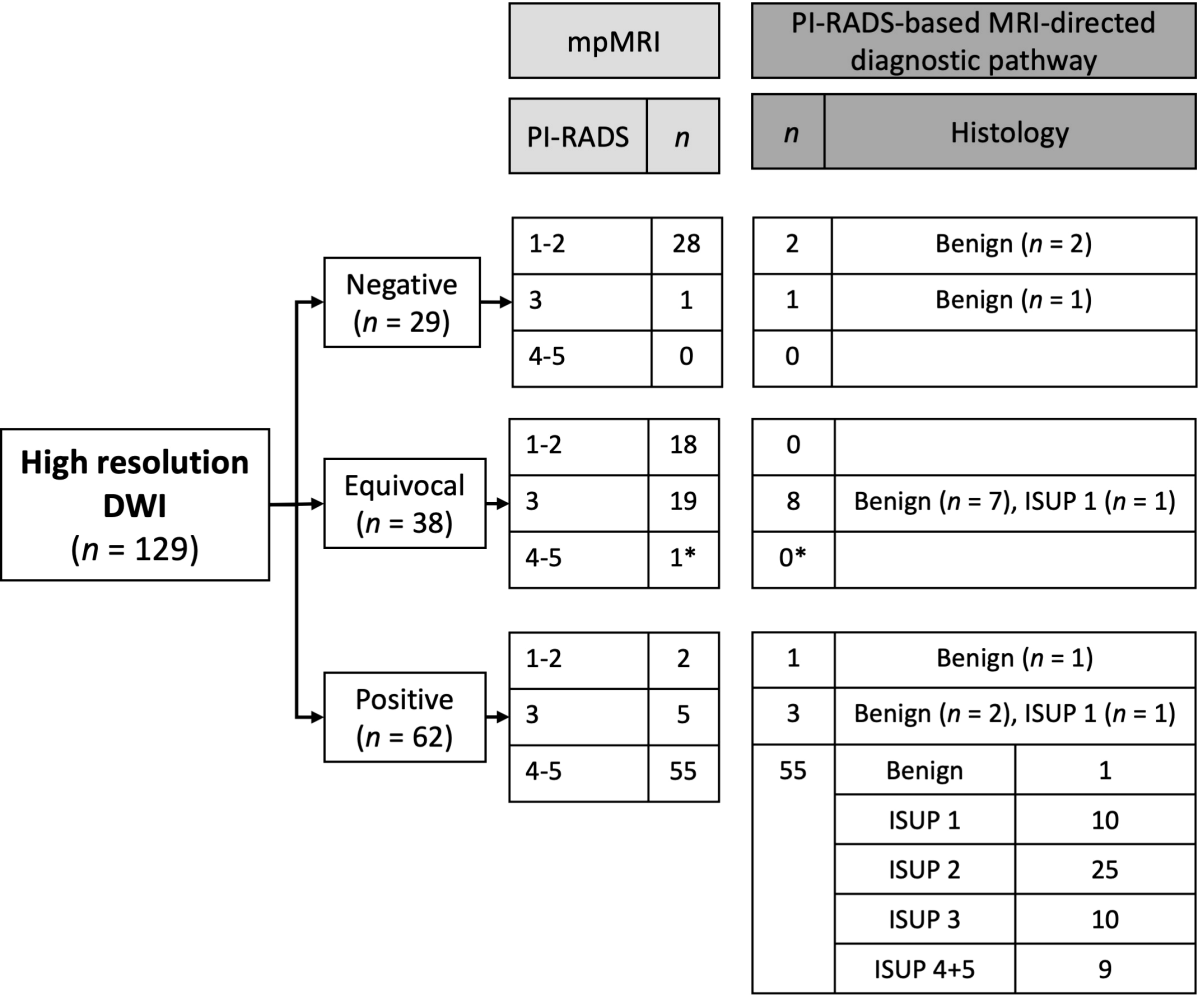
Results from the prospective interpretation of high-resolution DWI-only compared to PI-RADS (mpMRI) and biopsy results. *No biopsy performed because of comorbidity and a small lesion at MRI. The patient was referred to PSA monitoring and was not re-referred within the 55 months he was followed.

The findings from the prospective interpretations by the two primary readers are summarized in **Figure 4**. No patients were excluded due to insufficient image quality of the DWI.

Based on DWI-only 29 (22.5%) cases were categorized as negative, 38 (29.5%) as equivocal and 62 (48.1%) as positive. At mpMRI 48 (37.2%) cases were categorized as PI-RADS 1-2, 25 (19.4%) as PI-RADS 3 and 56 (43.4%) as PI-RADS 4-5. All PI-RADS 4-5 patients were categorized as positive at DWI-only, except one that was categorized as equivocal. This patient was not biopsied due to comorbidity and a small lesion at mpMRI. He was referred to PSA monitoring, has not been re-referred within the 55 months he was followed, and was considered to not have clinically significant cancer. Among the 62 patients categorized as positive at DWI-only, 7 (11.3%) were PI-RADS 1-3. Of the 67 negative or equivocal cases at DWI-only, 11 were biopsied as part of the PI-RADS based MRI-directed diagnostic pathway. No clinically significant cancers were detected. During the 56 months of safety net monitoring no non-biopsied patients were diagnosed with clinically significant prostate cancer. For the prospective readers the sensitivity, specificity, negative predictive value (NPV) and positive predictive value (PPV) of DWI-only to detect clinically significant cancer were 100.0%, 78.8%, 100.0%, and 71.0% (**Table 3**).

**Table 3:** 2x2 contingency table for detecting clinically significant prostate cancer for the prospective readers.

	DWI-only	
	Positive	Negative/Equivocal
ISUP ≥ 2 or cancer detected during follow-up	44 (TP)	0 (FN)
ISUP < 2 or no cancer detected during follow-up	18 (FP)	67 (TN)

**Figure 5:**
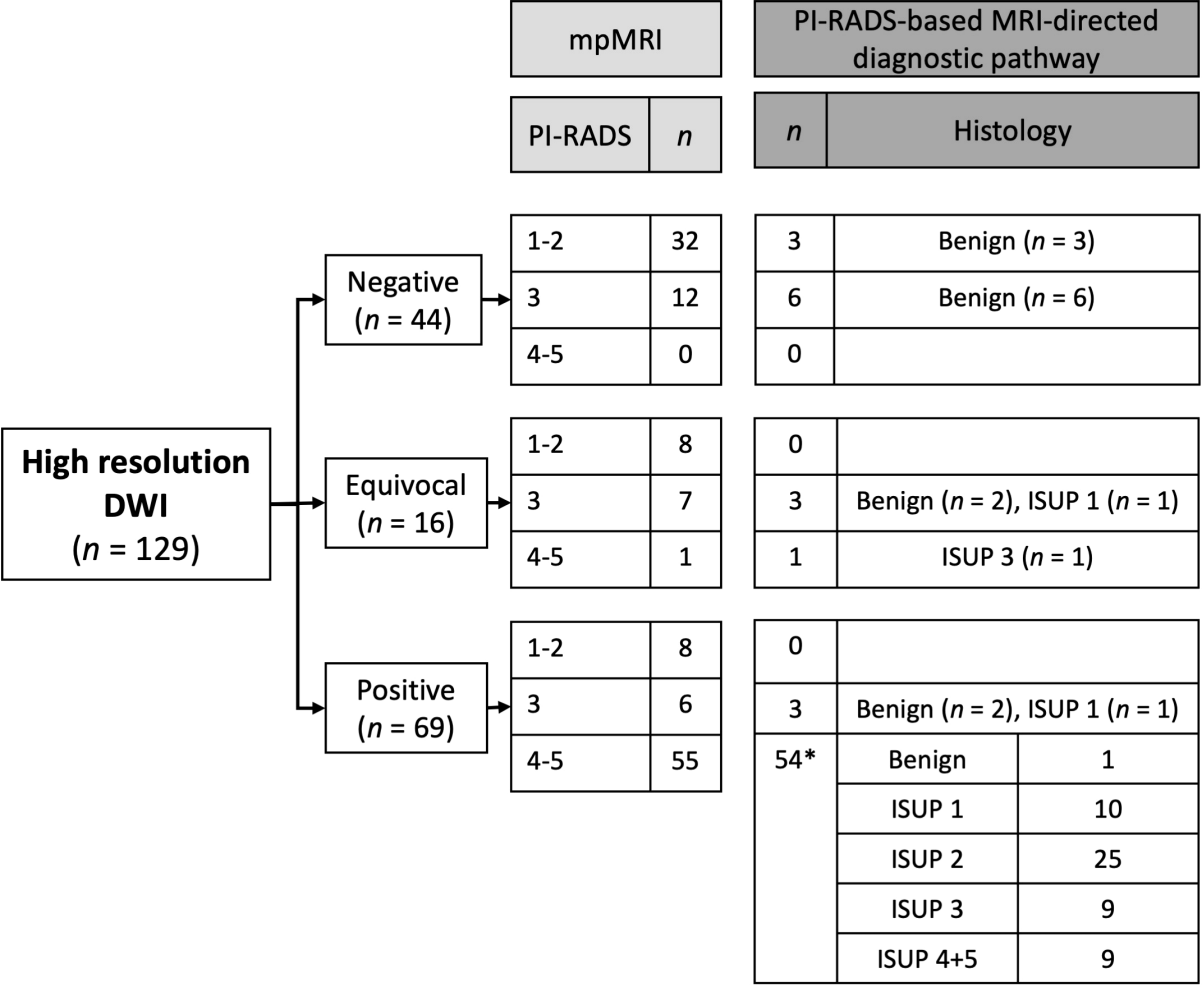
Results from the independent retrospective interpretation of high-resolution DWI-only compared to PI-RADS (mpMRI) and biopsy results. *No biopsy performed in one patient because of comorbidity and a small lesion at MRI. The patient was referred to PSA monitoring and was not re-referred within the 55 months he was followed.

The findings from the retrospective interpretations by the independent third reader are summarized in **Figure 5**. 44 (34.1%) of the DWI-only were categorized as negative, 16 (12.4%) as equivocal and 69 (53.5%) as positive. All PI-RADS 4-5 patients were categorized as positive at DWI-only, except one that was categorized as equivocal. Among the 69 patients categorized as positive at DWI-only, 14 (20.3%) were PI-RADS 1-3. Of the 60 negative or equivocal cases, 13 were biopsied as part of the PI-RADS based MRI-directed diagnostic pathway. One clinically significant cancer was detected in the equivocal group. For the retrospective reader the sensitivity, specificity, NPV, and PPV of DWI-only to detect clinically significant cancer were 97.7%, 69.4%, 98.3% and 62.3% (**Table 4**).

**Table 4:** 2x2 contingency table for detecting clinically significant prostate cancer for the retrospective reader.

	DWI-only	
	Positive	Negative/Equivocal
ISUP ≥ 2 or cancer detected during follow-up	43 (TP)	1 (FN)
ISUP < 2 or no cancer detected during follow-up	26 (FP)	59 (TN)

**Figure 6:**
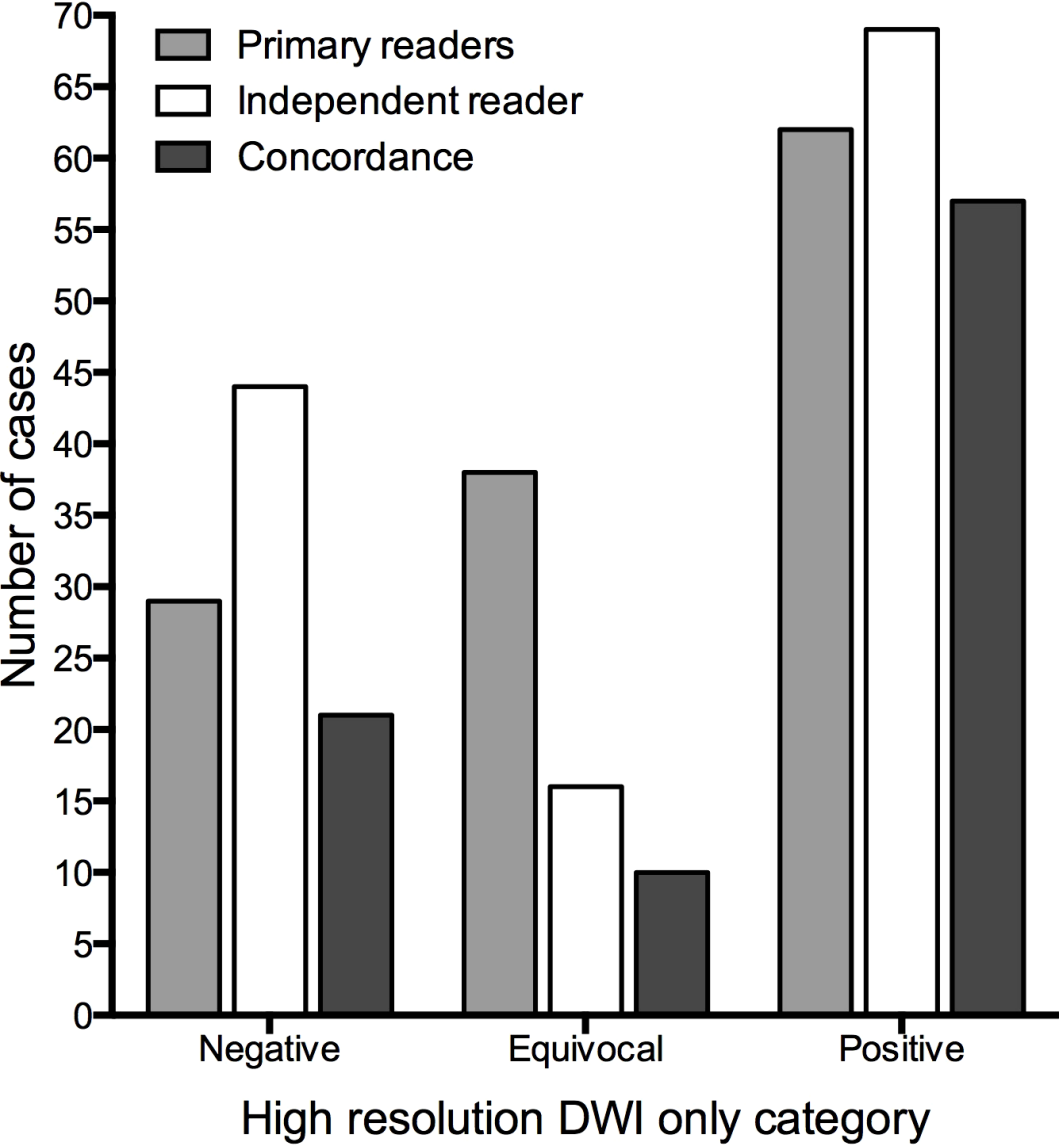
Comparison of the primary and the independent readings of high-resolution DWI-only.

**Figure 6** illustrates the agreement between the primary and the independent readings of DWI-only. There was high agreement on the interpretations categorized as positive, but less agreement on the negative and equivocal interpretations. The Cohen´s K for inter-reader agreement was 0.5 (p < 0.001).

**Figure 7:**
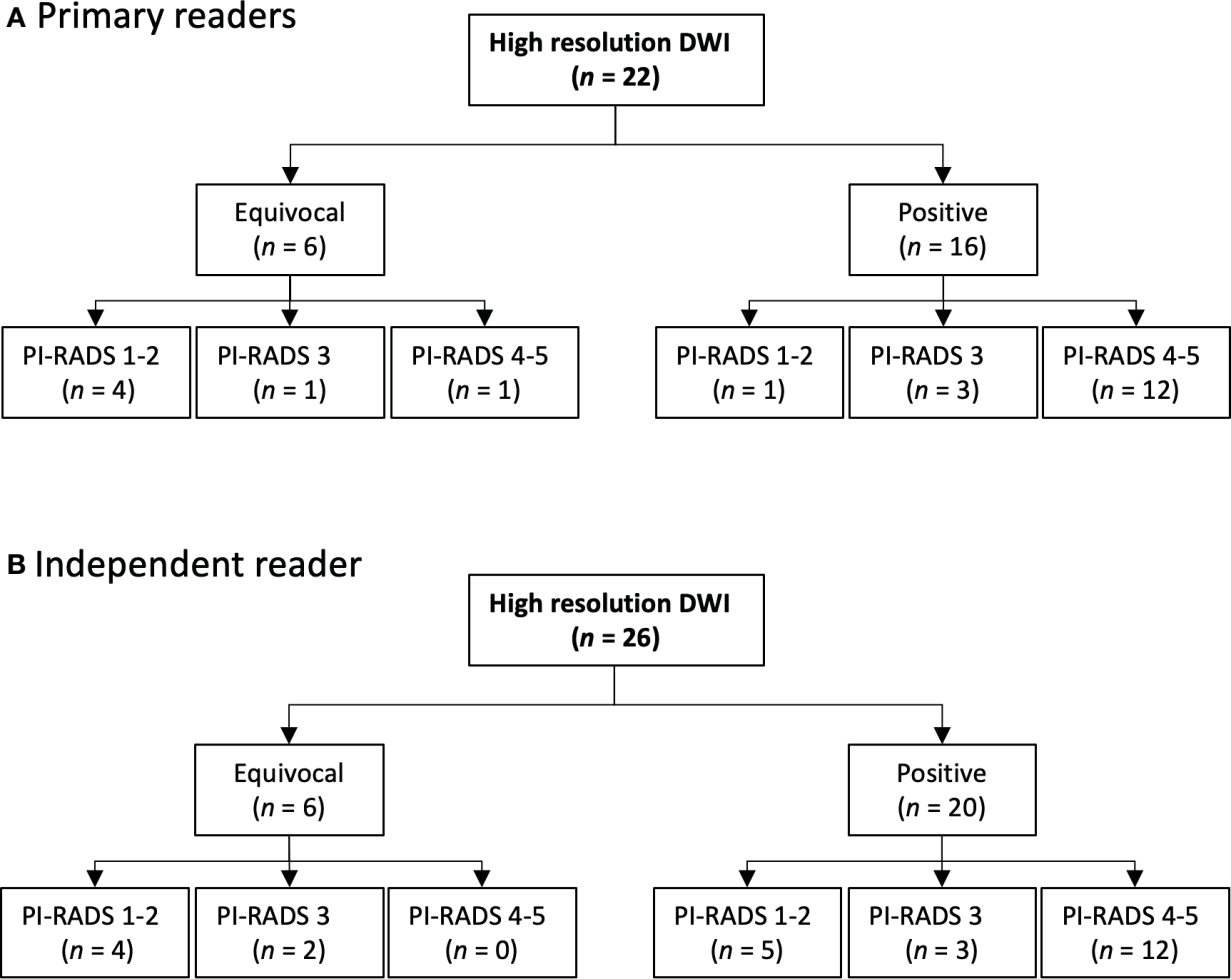
Transitional zone findings: Comparison of the primary and the independent readings of high-resolution DWI-only.

The findings at high-resolution DWI-only in the transitional zone were investigated separately. The primary readers detected and categorized 22 findings in the transitional zone and the independent reader 26. **Figure 7** shows the subsequent mpMRI-based PI-RADS score for the equivocal and positive interpretations of DWI-only in the transitional zone.

**Table 1** shows the demographic and clinical parameters for all patients and the three DWI-only categories of interpretation. There is a significant difference in PSA density between the three categories.

## 4 Discussion

There is growing interest in evaluating the feasibility of replacing mpMRI with bpMRI as a diagnostic approach for prostate cancer workup (3, 5, 15, 16). The PI-RADS steering committee has concerns about this and proposes an alternative approach in which patients are stratified to protocols with or without contrast medium based on clinical parameters (6). In this study we investigated if a monoparametric high-resolution DWI could identify patients with clinically significant cancer that might benefit from full mpMRI. The findings were compared to PI-RADS from mpMRI and the outcome of a PI-RADS based MRI-directed diagnostic pathway with 56 months follow-up. Using PI-RADS (mpMRI) as reference standard, all PI-RADS 4-5 were categorized as positive on DWI-only. There were no false negatives, but seven positives at DWI-only were PI-RADS 1-3 (11.3%). Using biopsy and safety net monitoring as reference standard, all ISUP ≥ 2 were categorized as positive on DWI-only, but 15 positives at DWI-only did not have clinically significant cancer (24.2%). Thus, the sensitivity and specificity of the DWI-only concept to detect clinically significant prostate cancer were 100.0% and 78.8%.

The results from several studies indicate that bpMRI and mpMRI have similar diagnostic performance in the detection of clinically significant cancer (3). We have not found other studies that have evaluated DWI-only in the work-up of patients with suspected prostate cancer, however, van der Leest et al. investigated a similar concept with a fast MRI protocol (5). They also acquired images only in the transversal plane, used sequential interpretation, and only three categories of likelihood of clinically significant cancer. The main difference is that they added T2W images to the DWI. Thus, their approach had better morphology (T2W) but lower spatial resolution in the DWI images. Despite this, their results were remarkably consistent with ours with high sensitivity for clinically significant cancer, but lower specificity. These results indicate that a fast MRI protocol without contrast medium can rule out clinically significant cancer for about half of the patients. In both studies, mpMRI was marginally better than the fast MRI approach to rule out clinically significant cancer.

The question arises how a fast MRI protocol best could be implemented in an MRI-directed diagnostic pathway. The PI-RADS committee’s narrative review states that implementing MRI without contrast medium must enhance operational benefit without compromising diagnostic performance (6). So, is the diagnostic performance of fast MRI sufficient to replace mpMRI? The results from our and other studies on detection of clinically significant cancer seem to suggest this (3). However, the debate seems to focus on detection and not staging. Prebiopsy MRI can be used for tumor staging and treatment planning as well (17, 18). Given the growth pattern of prostate cancer with the tendency of scattered tumor islets (19–21), an early phase fat-saturated DCE with high spatial resolution has, in theory, the best technical ability to detect tumor islets and thereby the tumor extension. In line with this, Caglic et al. found that DCE better detected seminal vesicle invasion (22) and Dinneen et al. found that DCE improved the performance of MRI in ruling out extra-prostatic extension (23). DCE has also been found to improve identification of some intra-prostatic tumors (24) and estimation of tumor volume (25). These observations suggest that DCE is important for precise assessment of the extent of the tumor. Therefore, an abbreviated MRI approach without DCE might compromise the diagnostic performance.

Could the concept of DWI-only as illustrated in **Figure 1** deliver operational benefits? An important prerequisite is that patients with equivocal findings/PI-RADS 3 do not immediately proceed to biopsy but are monitored, with PSA and/or serial MRIs. Among the patients categorized as equivocal at DWI-only in our study, none have been diagnosed with clinically significant cancer. There is no consensus on omitting biopsies in these patients. However, there are data indicating that this may be safe. Yerram et al. showed that equivocal lesions rarely harbor more than small foci of ISUP 2 (26) and there are other studies indicating that the cancers missed on MRI usually are low-grade, slow-growing and can be safely monitored (27–29). The data from our long-term safety net monitoring also contribute to the body of knowledge for such an approach.

In our clinical practice, DWI-only as a first step, with recall of positives to mpMRI, would have led to approximately 15% shorter scheduled examination time, 30% shorter scan time, and a 50% reduction in the use of contrast medium and anti-peristaltics, as compared to performing mpMRI in all patients. The design of an MRI-directed pathway is a trade-off between many considerations, as discussed by the PI-RADS steering committee (6).The approach with a fast MRI as a first step could reduce the need for mpMRI by about 50%, but with the disadvantage of an extra attendance for about half of patients. On-table monitoring, either by radiologist or by an artificial intelligence algorithm, could minimize the need for recalls, but would be logistically challenging (6). A major advantage of proceeding to mpMRI is precise assessment of the extent of the tumor, without image artifacts from biopsy, providing optimal treatment planning, whether it be focal treatment, surgery or radiotherapy. Furthermore, if sequences for detection of metastases are included, complete TNM-staging would be at hand for all patients with clinically significant cancer, without the need for further imaging.

The DWI-only concept may also be interesting for screening. It meets several of the suggestions that Eldred-Evans et al. discuss in order to optimize MRI for screening (9). We refined the DWI technique, and the protocol was less than 10 minutes. Furthermore, prostate volume enables calculation of PSA density, which is increasingly used to risk-stratify equivocal scan results before biopsy (30, 31). Interestingly, we found a clear association between PSA density and suspicious findings on the DWI-only.

In the transitional zone we anticipated to find false positives at DWI-only because we did not have the dominant T2W sequence to differentiate between tumors and hyperplastic nodules (PI-RADS). Interestingly, we found fewer than anticipated. The primary readers had only one positive finding at DWI-only that was PI-RADS 1-2 at mpMRI and the independent reader had five (**Figure 7**). A possible explanation is that, compared to conventional DWI, the high spatial resolution in the high-resolution DWI provides b0-images with sufficient T2-weighted morphological information to recognize hyperplastic nodules.

A strength of our study is that we used not only PI-RADS as a reference standard, but also biopsy results and 56 months of safety net monitoring, which would likely have detected false negative interpretations. Another strength is the third independent reader. This reader detected all except one of the clinically significant cancers, indicating that the results can be generalizable. We also used higher spatial resolution than specified in the PI-RADS guidelines. The PI-RADS steering committee emphasized that high image quality is of paramount importance as a part of routine unenhanced MRI (6). However, this could also be considered a limiting factor as it would require further changes to the current standard.

Our study has several limitations. It is a single center study, and the size of the study population is small. There are no validated criteria for interpretation of the DWI-only examination and importantly, we did not use saturation biopsies.

The idea of using DWI-only as a first step has some issues to consider: Patients whose examinations are affected by susceptibility artefacts from hip prostheses and/or rectal gas will need to proceed to/be recalled for mpMRI due to poor DWI quality. An important consideration is that some aggressive cancers have a diffuse infiltrative growth pattern and are therefore difficult to detect on DWI. However, such cancers are also difficult to detect on bpMRI. PSA-monitoring and mpMRI with DCE is probability the best approach to recognize that these patients must be biopsied. Finally, there will probably be operational benefits of implementing an initial DWI sequence as a first step in an MRI-directed pathway if the incidence of clinically significant cancer is low, but maybe not if the incidence is high.

## 5 Conclusions

In this concept study the monoparametric high-resolution DWI identified all patients with clinically significant cancer in a mpMRI-directed diagnostic pathway. Half of the patients had negative or equivocal findings at DWI-only, and 56 months of safety net monitoring did not reveal any significant cancers in these groups. These patients could have avoided further immediate work-up if DWI-only had been used as a first step. The other half might have benefited from proceeding to mpMRI providing optimal biopsy-guidance and treatment planning.

## Data availability statement

The raw data supporting the conclusions of this article will be made available by the authors, without undue reservation.

## Ethics statement

The studies involving human participants were reviewed and approved by The Norwegian Center for Research Data (982062). Written informed consent for participation was not required for this study in accordance with the national legislation and the institutional requirements.

## Author contributions

All authors made substantial contributions to the conception or design of the work, or the acquisition, analysis, or interpretation of data. JR, KH, UR and TS drafted and wrote the manuscript and JBM and IS revised it critically for intellectual content. All authors contributed to the article and approved the submitted version.
